# *Prevotella copri* facilitates wound healing in mice through the sphingosine-CerS1-ceramide metabolic pathway

**DOI:** 10.1128/spectrum.01587-25

**Published:** 2025-11-14

**Authors:** Meili Zhao, Yue Liu, Shuyao Lv, Taotao Mi, Nan Wang, Shuaishuai Zhang, Hailiang Liu

**Affiliations:** 1 Institute for Regenerative Medicine, State Key Laboratory of Cardiology and Medical Innovation Center, Shanghai East Hospital, School of Medicine, Tongji University 481875, Shanghai, China; 2 Institute of Advanced Biotechnology and School of Medicine, Southern University of Science and Technology 639321, Shenzhen, China; 3 Key Laboratory of Xinjiang Phytomedicine Resource and Utilization of Ministry of Education, College of Life Sciences, Shihezi University 675460, Shihezi, China; Tainan Hospital Ministry of Health and Welfare, Tainan, Taiwan

**Keywords:** *Prevotella copri*, Wound healing, Sphingosine, Microbiome-host interactions

## Abstract

Skin wound repair constitutes a sophisticated biological process involving spatiotemporally coordinated molecular cascades, with emerging evidence highlighting the dynamic regulatory role of skin microbiota. Utilizing a broad-spectrum antibiotic (ABX)-treated murine model, we identified *Prevotella copri* as a core functional commensal in the wound microecosystem that orchestrates tissue regeneration through metabolite-host crosstalk. ABX-induced microbial remodeling significantly enriched *P. copri* relative abundance, accelerated wound closure, and upregulated pro-regenerative factors vascular endothelial growth factor and epidermal growth factor. Metabolomic profiling revealed that *P. copri*-secreted sphingosine undergoes bioconversion to C18-ceramide via the non-canonical CerS1 pathway, driving keratinocyte hyperproliferation and neoangiogenesis. Pharmacological inhibition of CerS1 with P053 suppressed ceramide synthesis and delayed healing, mechanistically validating the sphingosine-CerS1-ceramide axis. Crucially, *P. copri* exhibits dual regulatory modalities: ecologically, β-lactamase-mediated antibiotic resistance establishes microbial dominance, while metabolically, sphingolipid-driven spatiotemporal signaling remodels the regenerative microenvironment. These findings align with and extend the evolving perspective of a functional wound microbiota and propose a potential synergistic strategy that combines targeted enrichment of beneficial commensals like *P. copri* with metabolic axis modulation to promote healing. Our findings elucidate a microecology-metabolism circuit that transitions wound management from passive anti-infection to precision intervention, providing a molecular blueprint for developing microbiome-reprogramming therapies in regenerative medicine.

**IMPORTANCE:**

Traditional wound repair research often focuses on microbial diversity, neglecting the critical role of specific taxa in tissue regeneration. Our study challenges this by highlighting *Prevotella copri* as a key species in wound healing, operating through the *Prevotella copri*-sphingosine-CerS1-ceramide signaling pathway. This discovery reshapes the understanding of microbiome-host interactions and paves the way for precision microbial therapies. By showing that a single bacterium can replace complex community dynamics, we connect ecological theory with regenerative applications, offering a strategy to use microbial metabolism for precise wound healing.

## INTRODUCTION

The skin, the largest organ of the human body, serves as a critical barrier against the invasion of external pathogens. Diverse microorganisms—including bacteria, fungi, and viruses—colonize the skin surface, forming a complex and dynamic microbiota. Notably, skin microbes, particularly those inhabiting wounds, rarely exist in a free-living planktonic state but instead assemble into structured biofilm communities (1–3). These biofilms confer enhanced resistance to environmental stressors and antimicrobial agents (4, 5), fundamentally shaping the ecological dynamics and functional impact of the skin microbiota.

*Prevotella copri*, an obligate anaerobe, persists on oxygen-exposed skin by occupying hypoxic niches such as deep hair follicles (>200 µm depth), where restricted oxygen diffusion creates localized anaerobic environments (6). Its survival is further facilitated through integration into polymicrobial biofilms, wherein oxygen consumption by peripheral bacteria maintains an anoxic core (4). Although *P. copri* is typically low in abundance on skin compared to the gut, its presence has been reliably detected via metagenomic sequencing, circumventing the limitations of aerobic cultivation (7).

Wound healing proceeds through four highly coordinated and overlapping phases: hemostasis, inflammation, proliferation, and remodeling. The process initiates with hemostasis, characterized by fibrin clot formation mediated by platelet activation (8, 9). This is followed by an inflammatory phase involving neutrophil and monocyte infiltration, which clears debris and primes the tissue for repair via tightly regulated cytokine signaling (10, 11). The proliferative phase is driven by three central mechanisms: re-epithelialization, facilitated by keratinocyte migration under the guidance of keratinocyte growth factor (KGF) and epidermal growth factor (EGF) (12–14); angiogenesis, stimulated by vascular endothelial growth factor (VEGF) and basic fibroblast growth factor, leading to new vessel formation (15–17); and matrix reconstitution, in which fibroblasts degrade the provisional matrix via matrix metalloproteinases and synthesize new extracellular matrix components such as type III collagen and proteoglycans (13, 18).

A subpopulation of fibroblasts differentiates into α-smooth muscle actin-positive myofibroblasts, which promote wound contraction (19, 20). During the final remodeling phase, type I collagen gradually replaces type III collagen, and the vasculature is refined through selective pruning (19, 21). Emerging evidence underscores the skin microbiota’s dual role in modulating healing. For instance, while *Staphylococcus aureus* can impair repair through the secretion of proteases and hemolysins (22), it also contributes to epithelial and neural regeneration via IL-1β-IL-1R-MyD88 axis and IL-17A/IL-17RA signaling, respectively (23, 24). Beneficial commensals support healing through multiple mechanisms, including TLR-mediated antimicrobial peptide induction (25–27), competitive suppression of pathogenic biofilms (28), short-chain fatty acid-dependent barrier reinforcement (29, 30), and immunomodulation via Treg/Th17 balance regulation (31).

These insights redefine the skin microbiota from a passive “pathogen reservoir” to a dynamic therapeutic interface. Current clinical practices—often reliant on indiscriminate microbial eradication—overlook functional heterogeneity among commensal species. To bridge this gap, developing spatiotemporal maps of microbial-host crosstalk and deciphering strain-specific mechanistic pathways—particularly regarding metabolite signaling—will catalyze precision microecological therapies. Such advances promise to revolutionize wound care, transitioning from empirical antimicrobial regimens to microbiota-reprogramming strategies that harness ecological resilience and metabolic virtuosity, ultimately reshaping the frontier of regenerative medicine.

## RESULTS

### Microbial regulation of cutaneous regenerative dynamics

**Fig 1:**
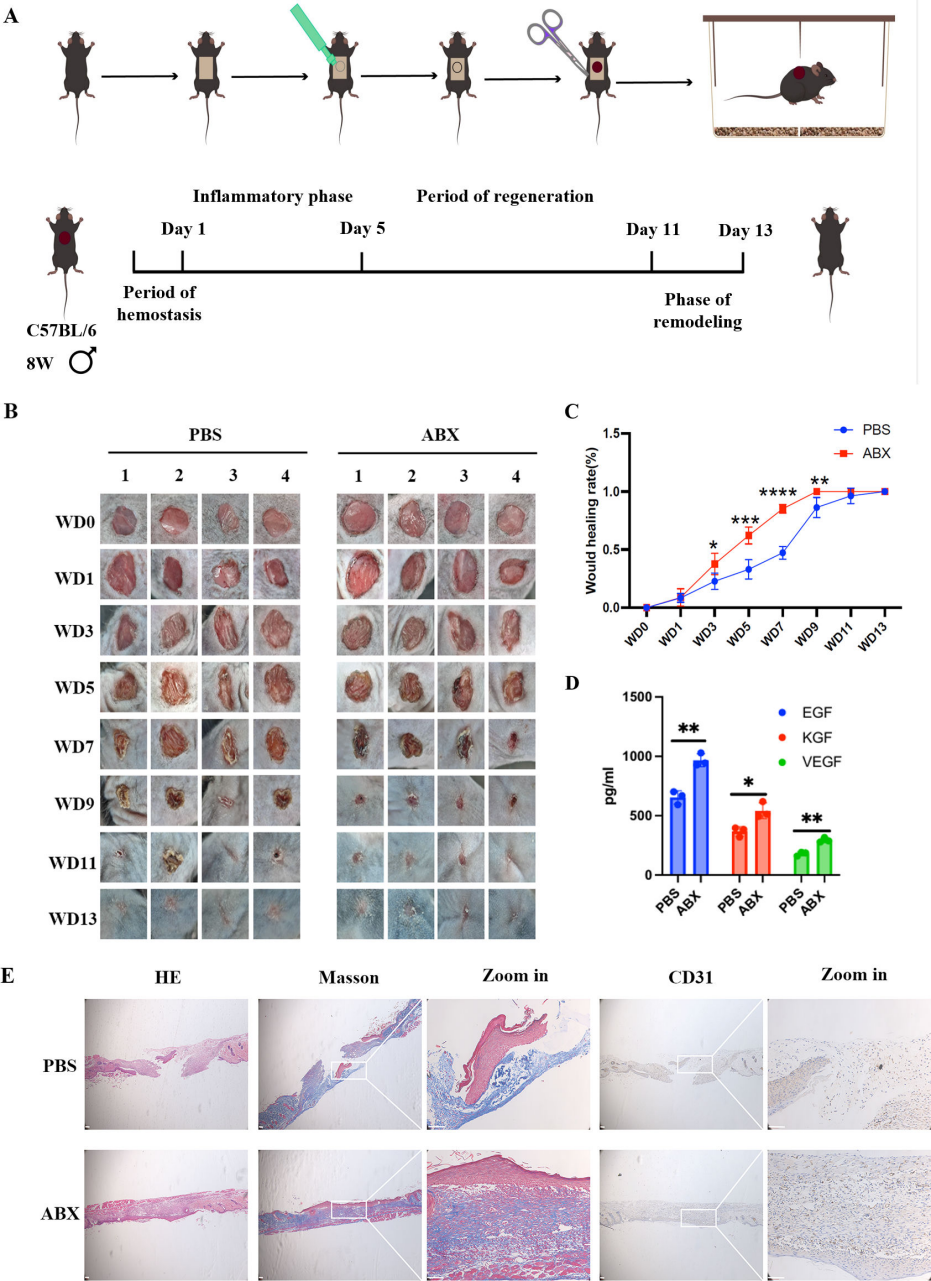
Microbial modulation of cutaneous wound healing dynamics. (**A**) Schematic workflow of the murine excisional wound model. (**B**) Representative macroscopic images of 6 mm excisional wounds in ABX and PBS control groups across post-wounding days 0–13 (*n* = 4, scale bar: 5 mm). (**C**) The ABX-treated group exhibited accelerated wound healing, achieving complete closure by day 8, whereas the PBS control group healed at a significantly slower rate. (**D**) ELISA quantification of pro-regenerative growth factors in wound tissues at day 8 post-injury. (**E**) Histomorphometric analysis of day 8 wound tissues: hematoxylin and eosin (HE) staining illustrating epidermal-dermal reorganization, Masson’s trichrome highlighting collagen architecture, and CD31 immunohistochemistry mapping neovascularization (scale bar: 50 µm). Data represent mean ± SEM; statistical significance between groups was determined by two-tailed Student’s *t*-test (*P ≤ 0.05, **P ≤ 0.01, ***P ≤ 0.001, ****P ≤ 0.0001).

To systematically investigate how microbiota regulates wound healing, we established a localized intervention model comparing broad-spectrum antibiotic (ABX)-treated wounds and PBS-treated controls (Fig. 1A). Wound healing assays revealed that ABX-induced microbial depletion significantly accelerated wound closure compared to controls (Fig. 1B and C). Transcriptomic analysis showed marked upregulation of pro-regenerative factors, including VEGF, EGF, and KGF, in the ABX group (Fig. 1D). Histological assessment further demonstrated enhanced tissue regeneration: hematoxylin and eosin (H&E) staining revealed restored epidermal-dermal integrity, and Masson’s trichrome staining showed increased and well-organized collagen deposition. In addition, immunohistochemical analysis of CD31 indicated markedly promoted neovascularization in ABX-treated wounds (Fig. 1E). Together, these results suggest that antibiotic-induced microbiota remodeling enhances wound repair through structural restoration, angiogenic activation, and upregulation of healing-promoting factors.

### Elucidating microbial drivers of accelerated wound healing

**Fig 2:**
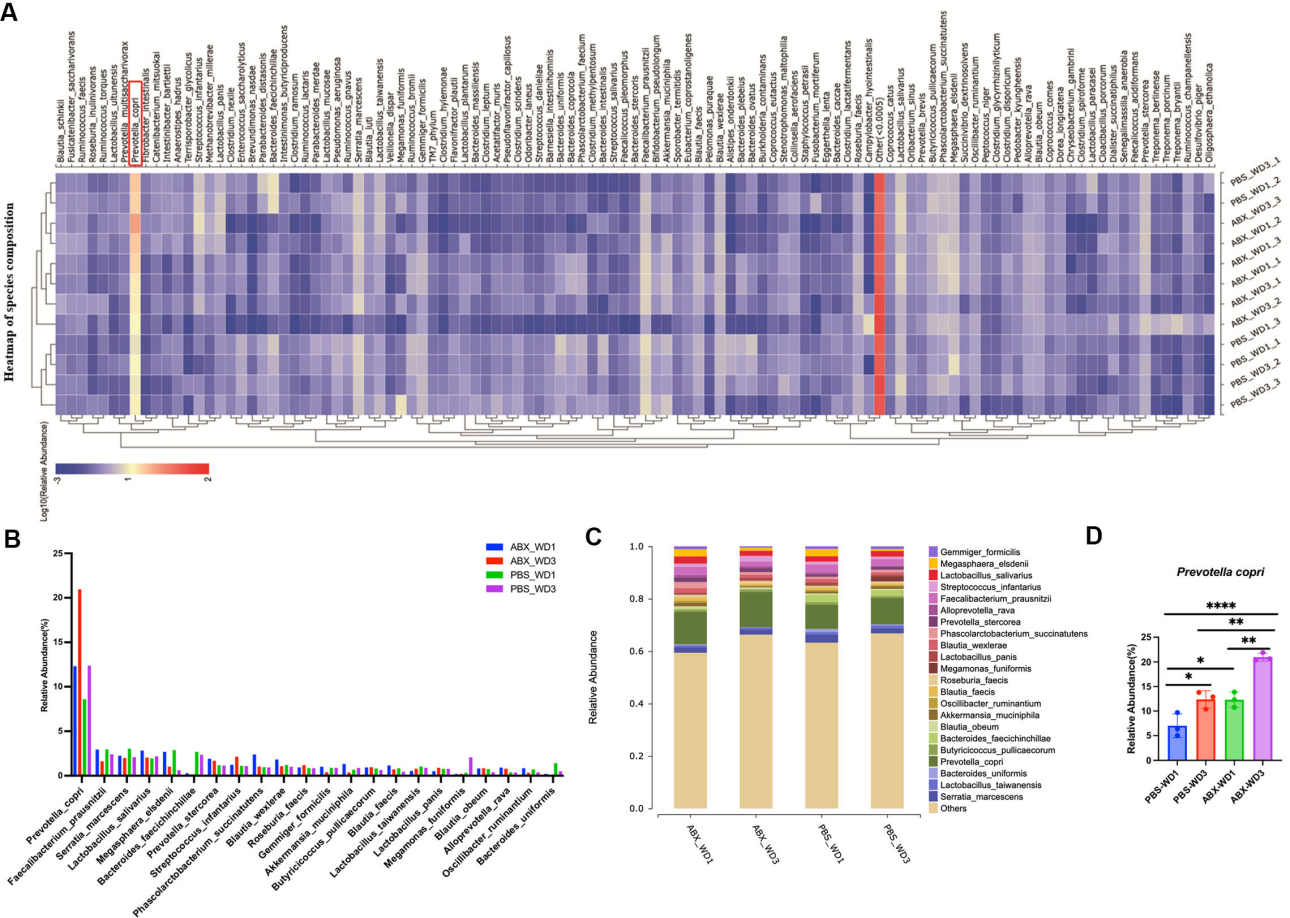
Ecological succession of wound microbiota driven by *Prevotella copri*. (**A**) Temporal heatmap depicting microbial community dynamics during the inflammatory phase at 24 and 72 h post-injury. (**B**) Comparative analysis of the most abundant taxa, highlighting *P. copri* as the most abundant species across experimental groups. (**C**) Taxonomic composition profiles illustrating microbial structural shifts between ABX and PBS control wounds. (**D**) Quantitative validation of *P. copri* abundance at critical time points, demonstrating its accelerated expansion in ABX-treated wounds versus gradual enrichment in controls. Data represent mean ± SEM; statistical significance was determined by two-tailed Student’s *t*-test (*P ≤ 0.05, **P ≤ 0.01, ****P ≤ 0.0001).

Building on the observed acceleration of wound healing following antibiotic treatment, we hypothesized that remodeling of the microbial community—rather than passive pathogen removal—constitutes a key mechanism enhancing repair in ABX-treated wounds compared to PBS controls. To test this, we performed spatiotemporal 16S rRNA sequencing of the wound microbiota at critical inflammatory time points (24 and 72 h post-injury; Fig. 2A). Taxonomic profiling revealed *Prevotella copri* as the most abundant taxon in both groups during early healing (Fig. 2A). Divergent colonization patterns were observed between treatments: PBS controls showed gradual enrichment of *P. copri*, consistent with natural repair progression, while ABX treatment triggered rapid expansion of *P. copri*, leading to its dominance within 72 h (Fig. 2B through D). This pattern suggests a density-dependent regulatory relationship, where *P. copri* biomass correlates with the activation level of host regenerative pathways.

These findings reposition *P. copri* as both an ecological organizer and a biochemical modulator in wound repair. Its colonization occurs in phases: initial niche establishment primes the wound microenvironment, followed by biomass accumulation that activates threshold-dependent healing mechanisms. This study moves beyond pathogen-centric perspectives and highlights microbial community engineering as a promising strategy for advancing regenerative therapies based on ecological principles.

### *Prevotella copri* enhances wound healing via sphingosine-mediated metabolic and structural reprogramming

**Fig 3:**
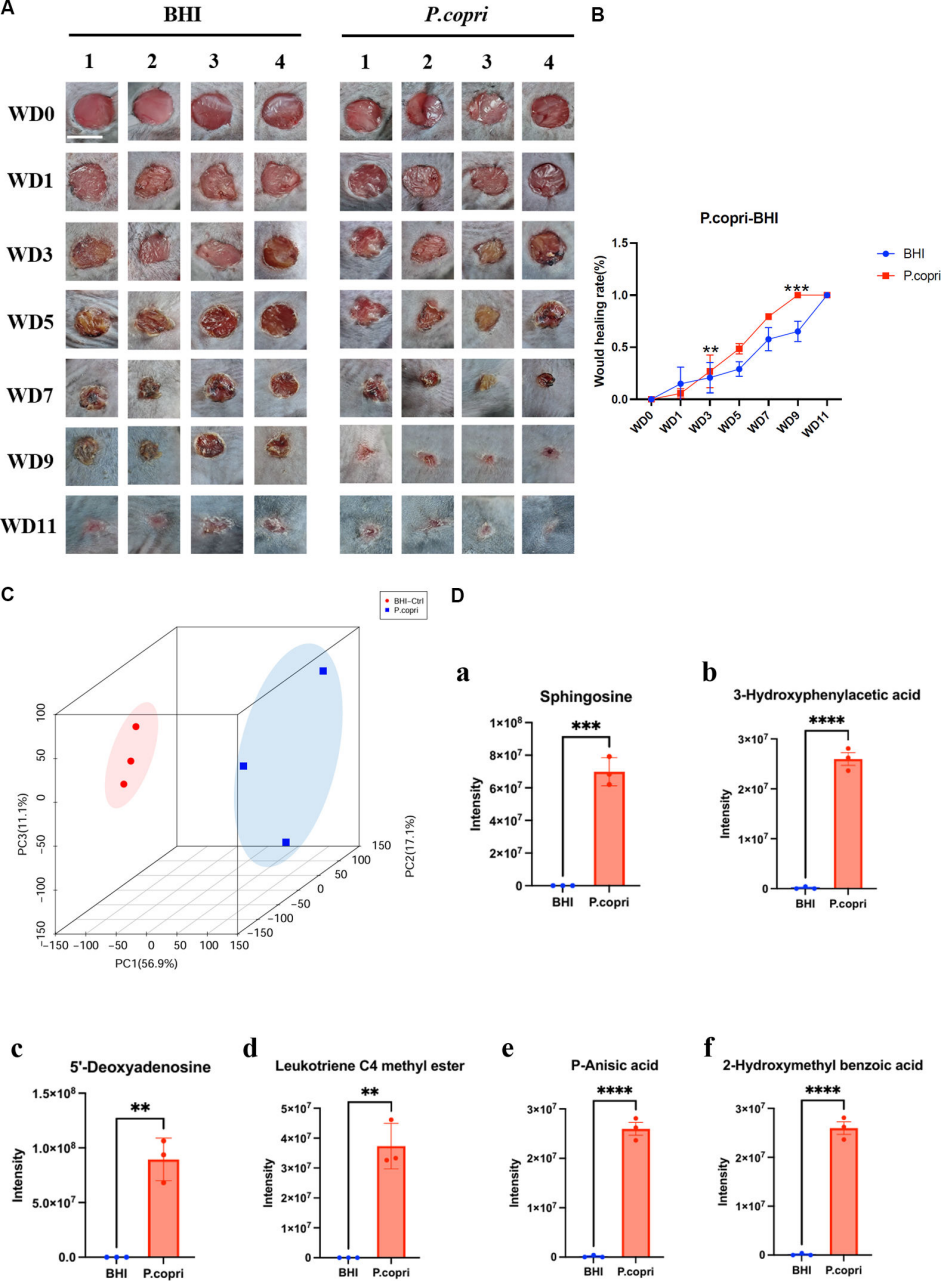
Therapeutic efficacy and metabolic signature of *Prevotella copri* secretions in wound healing. (**A**) Macroscopic progression of 6 mm excisional wounds treated with *P. copri* supernatant versus BHI medium controls, captured longitudinally from day 0 to 11 (*n* = 4, scale bar: 5 mm). (**B**) Kinetic profile of wound closure rates demonstrating accelerated re-epithelialization in the *P. copri* group. (**C**) Three-dimensional principal component analysis plot revealing distinct metabolic clustering between treatment groups. (**D**) Identified bioactive metabolites mediating therapeutic effects (relative quantification). (a) sphingosine (ceramide precursor), (b) 3-hydroxyphenylacetic acid (anti-inflammatory), (c) 5´-deoxyadenosine (energy metabolism modulator), (d) leukotriene C4 methyl ester (angiogenic mediator), (e) *p*-anisic acid (microbial antagonism), and (f) 2-hydroxymethyl benzoic acid (matrix remodeling cofactor). Data represent mean ± SEM; statistical significance was assessed by two-tailed Student’s *t*-test (**P ≤ 0.01, ***P ≤ 0.001, ****P ≤ 0.0001).

**Fig 4:**
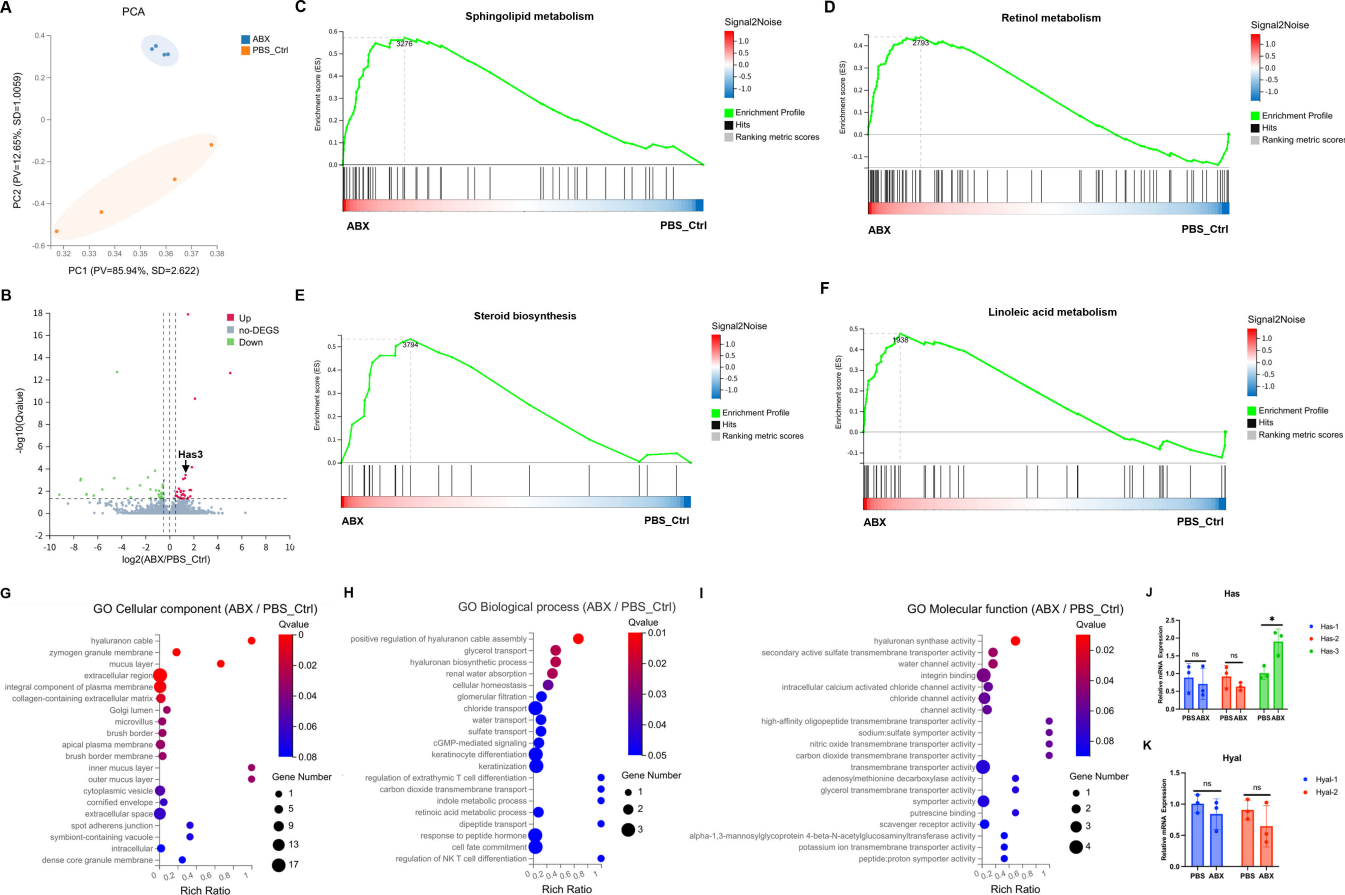
Metabolic reprogramming and hyaluronic acid remodeling in wound microenvironments. (**A**) Principal component analysis chart derived from RNA sequencing data. (**B**) Volcano plot illustrating differential gene expression. (**C–F**) Gene set enrichment analysis revealed systemic activation of pro-regenerative pathways in antibiotic-treated wounds, including sphingolipid metabolism, retinol metabolism, steroid biosynthesis, and linoleic acid metabolism. (**G–I**) GO term analysis demonstrated coordinated upregulation of hyaluronic acid (HA)-related functions: enriched HA-rich extracellular matrix organization (cellular component), enhanced HA fiber assembly (biological process), and elevated HA synthase activity (molecular function). (**J and K**) Quantitative PCR validation confirmed microbiome-driven HA homeostasis regulation, with ABX treatment significantly upregulating HA synthase Has3 expression (*P* < 0.01) while maintaining stable levels of HA degradation enzymes Hyal1 and Hyal2 (ns, non-significant). Data represent mean ± SEM; statistical significance was assessed by two-tailed Student’s *t*-test (*P ≤ 0.05, **P ≤ 0.01; ns, not significant).

To elucidate the molecular mechanisms underlying the pro-regenerative effects of *P. copri*, we topically applied its secreted metabolites (10⁹ CFU/mL) to murine wounds and observed significantly accelerated wound closure (Fig. 3A and B), indicating a metabolite-mediated effect. Metabolomic profiling revealed distinct biochemical signatures in *P. copri*-treated wounds, with sphingosine identified as a central bioactive molecule, accompanied by anti-inflammatory metabolites (*p*-anisic acid, 4-hydroxyphenylacetic acid) and angiogenesis-modulating compounds (e.g., leukotriene C4 methyl ester; Fig. 3C and D; Table S1). Transcriptomic analysis further indicated systemic metabolic reprogramming in antibiotic-treated wounds (Fig. 4A and B), characterized by coordinated upregulation of pathways involved in sphingolipid, retinol, and linoleic acid metabolism (Fig. 4C through F). Notably, *P. copri*-derived sphingosine was correlated with activation of host sphingolipid pathways, suggesting a cross-kingdom signaling mechanism through which bacterial metabolites directly stimulate endogenous repair processes.

Concurrently, hyaluronic acid (HA) matrix remodeling was identified as an additional regenerative mechanism. ABX treatment significantly enriched pathways related to HA metabolism (Fig. 4G through I) and specifically upregulated *Has3* (hyaluronic acid synthase), while expression levels of HA-degrading enzymes remained stable (Fig. 4J and K). This shift promoted net HA accumulation—a clinically established strategy for enhancing wound healing (32).

Together, these results support a dual mechanism whereby *P. copri* promotes wound repair through sphingosine-driven metabolic activation and HA-mediated matrix reinforcement, synergistically optimizing the wound microenvironment via microbial modulation of host biosynthetic networks. These findings position *P. copri* as a key microbial engineer capable of activating conserved mammalian repair pathways, highlighting the potential of targeted metabolite-based strategies to bridge microbial ecology and regenerative medicine.

### *Prevotella copri* drives wound healing via a non-canonical sphingosine-CerS1-ceramide axis

**Fig 5:**
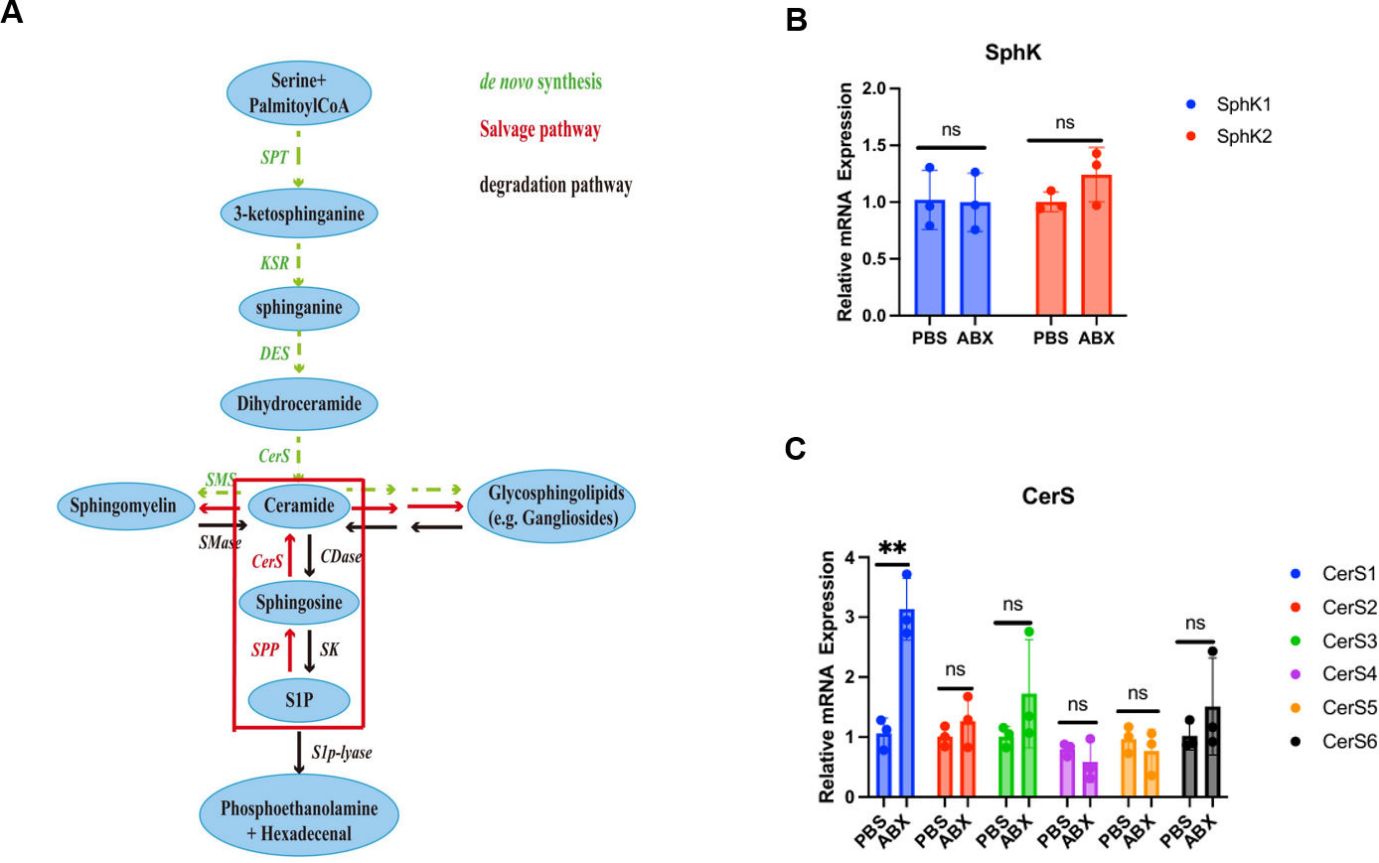
Non-canonical sphingosine metabolic rewiring in *Prevotella copri*-enhanced healing. (**A**) Schematic representation of sphingolipid metabolism pathways, highlighting the *P. copri*-mediated bypass of classical sphingosine phosphorylation. (**B and C**) qPCR validation of sphingosine pathway components in day 3 wounds: SphK1/2 expression remained stable (ns, non-significant), while ceramide synthase CerS1 exhibited 3.8-fold upregulation in ABX-treated wounds versus PBS controls (**P ≤ 0.01). Data represent mean ± SEM; statistical significance was assessed by two-tailed Student’s *t*-test.

Mechanistic investigation into the regenerative function of *Prevotella copri* revealed its specific ability to synthesize sphingosine, which activates host ceramide biosynthesis via CerS1-mediated metabolic reprogramming (Fig. 5A). Transcriptional and quantitative PCR (qPCR) analyses of sphingolipid pathway genes showed selective upregulation of *CerS1* in ABX-treated wounds, while sphingosine kinases (*SphK1/2*) remained unchanged—a pattern indicative of non-canonical sphingosine signaling (Fig. 5B and C). This “downstream activation, upstream stabilization” profile suggests that microbially derived sphingosine bypasses typical phosphorylation routes and directly promotes CerS1-driven ceramide production.

**Fig 6:**
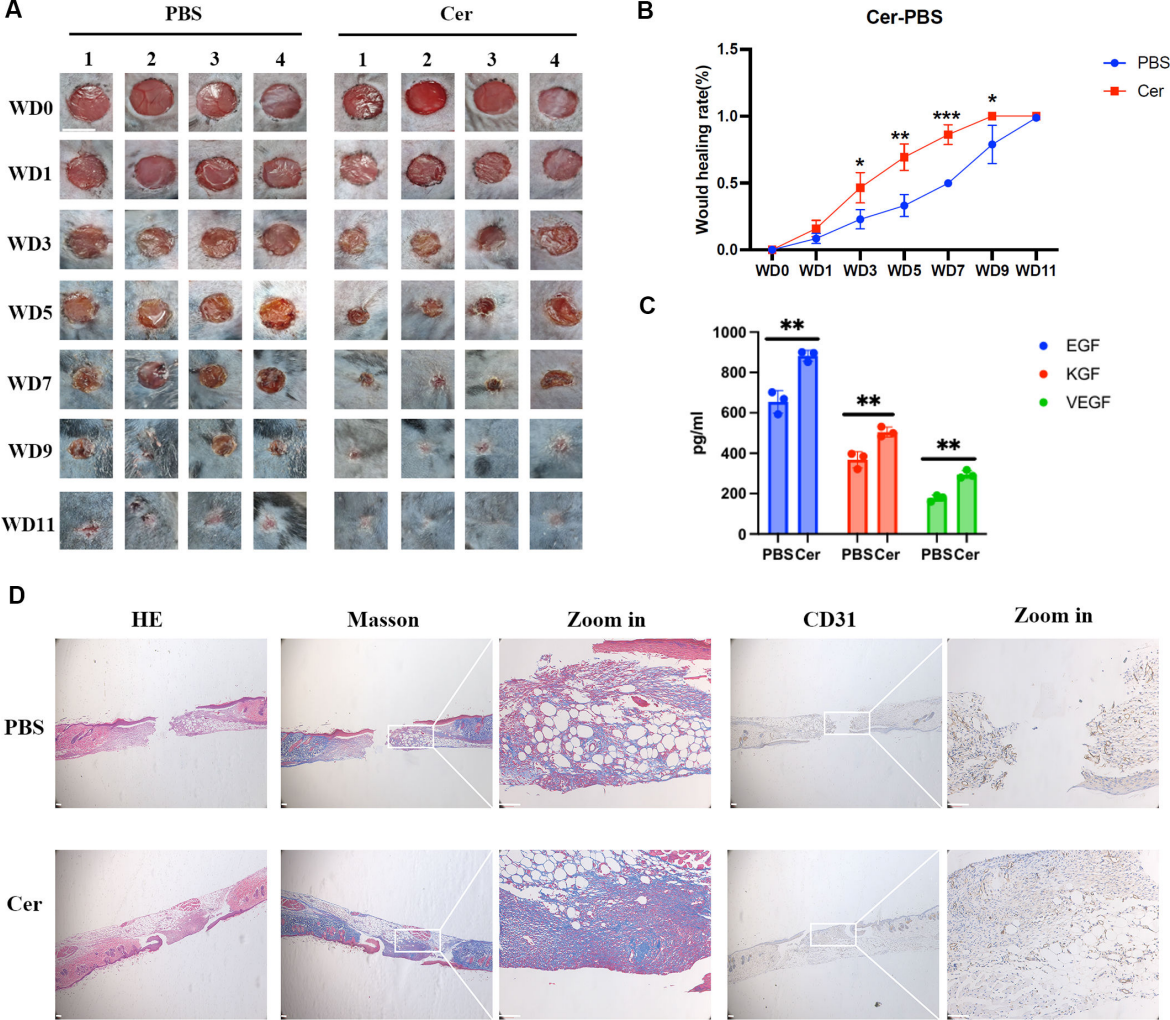
Ceramide accelerates wound healing through structural and molecular remodeling. (**A**) Macroscopic progression of 6 mm excisional wounds treated with 0.781 µM ceramide (Cer) versus PBS controls, captured longitudinally from day 0 to 11 (*n* = 4, scale bar: 5 mm). (**B**) Kinetic profile of wound closure rates demonstrating Cer-mediated healing acceleration (ceramide dose: 0.781 µM). (**C**) ELISA quantification of pro-regenerative factors at day 8 post-injury, showing Cer-induced upregulation of EGF, KGF, and VEGF. (**D**) Histomorphometric analysis of day 8 tissues: hematoxylin and eosin (HE) staining revealing enhanced epidermal-dermal junction reconstruction, Masson’s trichrome demonstrating increased collagen density, and CD31 immunohistochemistry confirming elevated neovascularization (scale bar: 50 µm). Data represent mean ± SEM; significance was determined by two-tailed Student’s *t*-test (*P ≤ 0.05, **P ≤ 0.01, ***P ≤ 0.001).

Functional validation confirmed the therapeutic role of ceramide: topical application significantly accelerated wound closure (Fig. 6A and B) and upregulated expression of EGF, KGF, and VEGF (Fig. 6C). Histological examination revealed enhanced restoration of the epidermal-dermal junction, increased collagen density, and promoted neovascularization, supporting ceramide’s role as the central effector molecule in *P. copri*-mediated repair (Fig. 6D).

**Fig 7:**
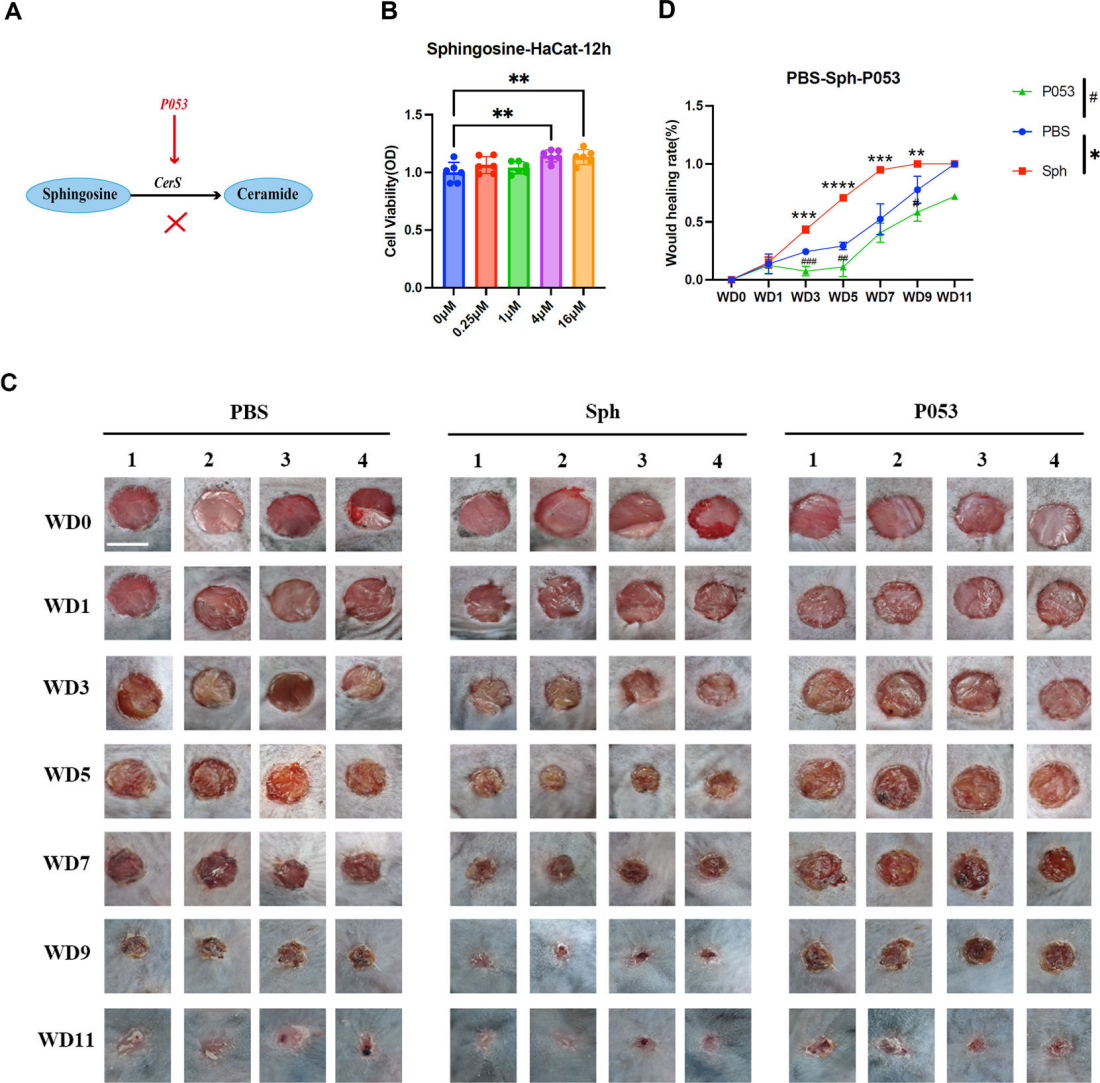
Pharmacological validation of the sphingosine-ceramide axis in wound repair. (**A**) Mechanistic schema illustrating P053-mediated inhibition of CerS1-driven ceramide biosynthesis, disrupting the *Prevotella copri*-initiated regenerative cascade. (**B**) CCK-8 assay revealed sphingosine-induced keratinocyte hyperproliferation (1.9-fold, *P* < 0.001) within 12 h, confirming its direct mitogenic activity. (**C**) Macroscopic progression of 6 mm wounds treated with sphingosine (4 µM) or co-administered with the CerS1 inhibitor P053 (1 µM), demonstrating complete abolition of healing acceleration by pathway blockade (*n* = 4, scale bar: 5 mm). (**D**) Time-course quantification of closure rates: sphingosine monotherapy enhanced healing, while P053 co-treatment reverted kinetics to baseline levels. Data represent mean ± SEM; significance was determined by two-tailed Student’s *t*-test (**P ≤ 0.01, ***P ≤ 0.001, ****P ≤ 0.0001).

Critically, the necessity of CerS1 was established through pharmacological inhibition: co-administration of sphingosine with the CerS1 antagonist P053 completely abolished the healing acceleration, whereas sphingosine alone sustained pro-regenerative effects (Fig. 7A through D). This combination of gain-of-function (ceramide supplementation) and loss-of-function (CerS1 inhibition) definitively identifies the sphingosine-CerS1-ceramide axis as the core mechanism underlying the therapeutic action of *P. copri*.

The discovery of this microbial-metabolic bypass reveals an evolutionary convergence between bacterial metabolite production and host lipid signaling pathways. By precisely delivering bioactive metabolites that modulate conserved host repair pathways, *P. copri* exemplifies how microbiota can functionally augment endogenous regenerative networks, offering new avenues for microbiome-based therapeutic strategies.

## DISCUSSION

### Ecological and functional role of *P. copri* in wound healing

As a keystone symbiont within the skin wound microecosystem, *Prevotella copri* exhibits unique ecological and functional specialization in orchestrating tissue regeneration. This anaerobic bacterium thrives in both ABX and PBS wounds, leveraging β-lactamase-mediated antibiotic resistance to establish ecological dominance—a trait that ensures its survival under antimicrobial pressure. Longitudinal analysis revealed its intrinsic repair role: while ABX intervention triggered rapid *P. copri* proliferation peaking at 72 h post-injury, control wounds displayed progressive enrichment, underscoring its fundamental contribution to physiological healing beyond mere antibiotic selection. This “competitive release” dynamic—where microbiota restructuring alleviates ecological constraints—propels *P. copri* into a leadership role, accelerating repair through prioritized niche colonization.

The antibiotic-induced dominance of *P. copri* represents an artificial monoculture resulting from broad-spectrum depletion of competitors (e.g., vancomycin/neomycin eliminating gram^+^/gram^−^ facultative anaerobes), which bypasses natural wound succession, where anaerobes like *Prevotella* gradually emerge in hypoxic niches within polymicrobial biofilms (6).

### Comparative mechanisms among commensals

While *Prevotella copri* is a well-established gut commensal (33), its detection on skin, though low in abundance, highlights the metabolic flexibility of this anaerobe and the capacity of skin microenvironments to support transient colonization. Our findings that *P. copri* promotes repair add to a growing list of beneficial wound microbes, including *Staphylococcus epidermidis* (34) and *Lactobacillus rhamnosus* GG (35–37). Notably, the mechanism we propose—dependent on bacterially derived sphingosine—is distinct from the sphingomyelinase activity of *S. epidermidis* or the lysate-driven migration induced by *L. rhamnosus*, underscoring the diverse strategies microbes employ to influence host repair. Furthermore, it is well-established that different commensals employ distinct mechanisms to interact with the host. For instance, *Bacteroides fragilis* modulates host immunity through polysaccharide A via the TLR2 pathway (38), whereas our results support that *P. copri* facilitates wound healing through a unique sphingolipid-mediated metabolic pathway.

### Sphingosine-mediated mechanism and divergent microbial strategies

Our findings that *P. copri*-derived sphingosine accelerates wound healing present a fascinating parallel and point of divergence from the work of Zheng et al. (34), who demonstrated that commensal *Staphylococcus epidermidis* secretes a sphingomyelinase (ScpA) that hydrolyzes host-derived sphingomyelin into ceramides to fortify the epidermal permeability barrier in homeostatic skin. This comparison reveals that distinct commensals have evolved divergent strategies to modulate host lipid biology: *S. epidermidis* functions as an “enzymatic modifier” by processing host lipids on the skin surface, whereas *P. copri* acts as a “*de novo* synthesizer” of bacterial sphingosine within deep hypoxic niches like hair follicles and wound beds. Consequently, their physiological impacts are context-specific; while *S. epidermidis* primarily enhances structural barrier integrity, *P. copri* predominantly promotes dynamic tissue repair through stimulating keratinocyte migration and re-epithelialization.

### The ceramide-microbiota axis across tissues

The intriguing interplay between microbiota and ceramide signaling is an emerging theme in host-microbe biology, particularly in the gut (39). Our discovery of a *P. copri*-ceramide axis in skin wound healing invites a comparative discussion of shared and unique features of this pathway across organ systems. Several shared features are notable: commensal bacteria serve as a significant source of sphingolipids in both gut and skin environments, with Bacteroidetes (e.g., *Bacteroides* spp.) being major producers in the gut (40), analogous to our findings involving the Bacteroidetes member *P. copri* on skin. Moreover, ceramides and their metabolites—such as sphingosine-1-phosphate (S1P)—from both sources are likely to engage conserved host signaling pathways (e.g., S1P receptors) to modulate fundamental cellular processes including epithelial proliferation and immune cell migration.

However, important differential features exist. First, the functional outcome is starkly different: bacterial ceramides in the gut are often associated with metabolic dysfunction and inflammation (39, 41), whereas *P. copri-*derived sphingosine demonstrates pro-healing and anti-inflammatory roles in acute skin wounds. Second, the microenvironments differ substantially; the gut constitutes a nutrient-rich, chronically antigenic setting where ceramide signaling frequently contributes to barrier dysfunction during dysbiosis, whereas the skin wound represents a site of active repair and transient inflammation in which localized ceramide/sphingosine delivery may preferentially promote re-epithelialization and inflammation resolution. Finally, the specific molecular species of bacterial sphingolipids (e.g., differing in chain length and saturation) may vary across bacterial genera and strains, potentially leading to divergent host responses—the unique structure of *P. copri*-derived sphingolipids could thus be a key determinant of its beneficial effects on skin.

### Integrated healing mechanism and therapeutic implications

The bacterium’s therapeutic efficacy arises from a dual survival-repair strategy. Ecologically, β-lactamase genes create microbial sanctuaries, enabling persistence amidst antimicrobial challenges. Metabolically, *P. copri* synthesizes sphingosine, which activates the CerS1-ceramide axis to directly stimulate keratinocyte proliferation and neovascularization. This synergy of ecological resilience and metabolic signaling redefines *P. copri*: no longer just an opportunistic colonizer, it acts as a conductor of active repair, adept at dominating both disturbed and natural microenvironments by adapting its role to promote tissue regeneration.

Clinically, these insights pioneer two transformative approaches: ecological engineering to enhance *P. copri* colonization through competitive microbiome modulation and metabolite-based therapies utilizing sphingosine or stabilized ceramide derivatives. By bridging microbial ecology with precision metabolic intervention, this work shifts wound management from passive antimicrobial regimens to active microecosystem reprogramming, offering a blueprint for microbiome-guided regenerative medicine. The ability of a single commensal species to functionally replace complex community interactions challenges conventional paradigms, opening new frontiers in chronic wound therapy and host-microbe interface engineering.

### Limitations and methodological considerations

The role of antibiotics in wound healing is complex and context-dependent. While some regimens, particularly targeted topical applications, can improve outcomes by reducing pathogen load or via direct anti-inflammatory effects (42), broad-spectrum systemic antibiotics often disrupt microbial ecology and impair healing. Our model, which examines recovery from such disruption, reveals that the specific compositional shifts post-antibiotics are critical: enrichment of a beneficial commensal like *P. copri* can rescue healing, whereas dominance of pathobionts would likely exacerbate delay.

Our reliance on bacterial conditioned media (CM), rather than viable *P. copri*, represents a deliberate strategy to isolate secreted bioactive factors; however, this approach also presents certain limitations. While effective for identifying soluble mediators such as sphingosine, the CM method may overlook contributions from non-secreted or cell-associated components—such as LPS—which could elicit divergent immune and metabolic responses. LPS derived from *P. copri* is of particular interest: although typically pro-inflammatory, its effects are highly context-dependent and may contradict the pro-regenerative outcomes observed in this study. Thus, the absence of whole-cell or specific component treatments precludes a comprehensive understanding of the full scope of *P. copri*-host interactions.

We cannot rule out the possibility that other species may also be involved and produce sphingolipid metabolites. In the future, we can use correlation analysis between species and metabolites to clarify the relationship and then verify it using germ-free mice.

### Future directions

While our CD31 data demonstrate enhanced angiogenesis, a key component of wound healing, future studies will benefit from the inclusion of specific immunohistochemical markers such as keratin 14 to directly quantify re-epithelialization dynamics.

A more detailed kinetic analysis beyond the endpoint assessment would provide deeper insights into the healing dynamics. While the present study focused on the standard 7-day post-wounding time point—commonly used to evaluate mature granulation tissue and angiogenesis in murine models—future studies incorporating multi-time-point analyses will be essential to fully elucidate the temporal sequence and regulatory mechanisms underlying the pro-healing effects induced by *P. copri* metabolites.

The exclusive use of male mice in this study minimized the confounding effects of hormonal fluctuations associated with the estrous cycle (43); however, future investigations in female models are essential to confirm the generalizability and sexual dimorphism of the observed healing effects.

While our murine data establish a proof-of-concept mechanism, a critical future goal will be to test the effect of *P. copri*-derived sphingosine on wound healing in *ex vivo* human skin models and primary human keratinocytes to confirm its translational potential.

Future studies employing genetically engineered *P. copri* strains will be crucial to definitively establish the role of bacterial ceramide synthesis in mediating host-microbe interactions *in vivo*. To further validate the causal contribution of *P. copri* to wound healing, key genes involved in glycosphingolipid metabolism could be knocked out via genetic editing, followed by functional assessment of wound repair outcomes. Additionally, the development of bacteriophage-based targeting strategies against *Prevotella* could offer a selective microbial clearance tool to corroborate its therapeutic function. Complementing these approaches, the use of purified microbial molecules—such as LPS and outer membrane vesicles—will help dissect their individual contributions and clarify whether their effects align with or antagonize the pro-healing activities observed in this study.

## MATERIALS AND METHODS

### Establishment of a mouse skin injury model

Eight-week-old male C57BL/6 mice were anesthetized using avertin (250 mg/kg, intraperitoneal injection) and subjected to dorsal hair removal with electric clippers. Full-thickness excisional wounds (6 mm diameter) were created using a sterile biopsy punch (RWD Life Science) and surgical scissors to ensure complete removal of epidermal and dermal layers. Wound progression was documented daily under standardized imaging conditions with a fixed camera height and LED ring light. Mice were individually housed in ventilated cages and received daily topical treatments. Wound closure rates were quantified using ImageJ software by calculating the percentage reduction in wound area relative to the initial size. Only male mice were used in this study to minimize variability in wound healing rates and immune responses influenced by the fluctuating estrogen levels of the estrous cycle in females (43).

### Preparation of ABX solution

Topical administration of the classical quadruple antibiotic regimen—comprising ampicillin (β-lactam), vancomycin (glycopeptide), metronidazole (nitroimidazole), and neomycin (aminoglycoside)—was employed to broadly deplete commensal microbiota. This combination synergistically targets diverse bacterial groups: ampicillin eliminates gram^+^/gram^−^ facultative anaerobes but inherently enriches β-lactamase-producing strains like *Prevotella copri*, which expresses chromosomally encoded cfxA penicillinases. Vancomycin suppresses gram^+^ competitors (e.g., Firmicutes), while neomycin depletes gram^−^ facultative anaerobes, indirectly promoting anaerobic expansion. Metronidazole exerts pressure on obligate anaerobes, though ~30% of *P. copri* strains evade this via *nim* gene-mediated resistance. Ampicillin (A5354), vancomycin (V0045000), metronidazole (M3761), and neomycin (33492) were procured from Sigma-Aldrich (St. Louis, MO, USA). The ABX solution was composed of 100 mg/kg ampicillin, 50 mg/kg vancomycin, 100 mg/kg metronidazole, and 100 mg/kg neomycin, administered twice daily by syringe drip.

### ELISA detection of growth factors

Skin tissues from designated time points were homogenized in RIPA buffer containing protease inhibitors (Beyotime) and centrifuged to obtain supernatants. Growth factor concentrations (VEGF, EGF, and KGF) were measured using species-specific ELISA kits (Jiangsu Jingmei Biotech) according to manufacturer protocols. Absorbance at 450 nm was recorded with a microplate reader (SpectraMax iD5, Molecular Devices), and values were interpolated from standard curves.

### Tissue processing and paraffin embedding

Excised wound tissues were fixed in 4% paraformaldehyde for 24 h at 4°C, followed by sequential dehydration in graded ethanol solutions (70%, 80%, 90%, 95%, 100%), clearing in xylene, and paraffin embedding using a histology embedding station (Leica EG1150H). Paraffin blocks were sectioned at 5 µm thickness with a rotary microtome (Leica RM2235), mounted on charged slides, and dried at 42°C for subsequent staining.

### H&E staining

Deparaffinized sections were rehydrated through xylene and graded ethanol series. Nuclei were stained with Harris hematoxylin for 5 min, followed by cytoplasmic counterstaining with eosin Y for 1 min. Sections were dehydrated in ethanol, cleared in xylene, and coverslipped with resinous mounting medium. Tissue architecture was analyzed using bright-field microscopy.

### Masson’s trichrome staining

Following deparaffinization and rehydration, sections were stained with Weigert’s hematoxylin for 10 min to visualize nuclei. Subsequent immersion in Ponceau S/acid fuchsin solution for 5 min highlighted connective tissue, followed by differentiation in 1% phosphomolybdic acid. Collagen fibers were selectively stained with aniline blue for 5 min, and sections were dehydrated, cleared, and mounted for analysis of matrix deposition patterns.

### Immunohistochemical staining

Antigen retrieval was performed by heating sections in citrate buffer (pH 6.0) at 95°C for 20 min. Endogenous peroxidase activity was quenched with 3% hydrogen peroxide. After blocking with 3% BSA (Solarbio), sections were incubated overnight at 4°C with anti-CD31 primary antibody (1:200, Abcam), followed by HRP-conjugated secondary antibody (DAKO) for 30 min. DAB chromogen (DAKO) was applied for signal development, and nuclei were counterstained with Mayer’s hematoxylin. Sections were imaged using a high-resolution slide scanner (3DHISTECH PANNORAMIC 250) at 20× magnification.

### Microbial sampling and 16S rRNA sequencing analysis of cutaneous wounds

To characterize the wound microbiota dynamics, sterile cotton swabs were rigorously rotated 60 times across wound beds at days 1 and 3 post-injury, ensuring comprehensive microbial collection. Swabs were immediately transferred to pre-chilled cryotubes, homogenized in sterile PBS with vortexing, and flash-frozen at −80°C until processing. Microbial genomic DNA was extracted using the MagPure Stool DNA KF Kit B (Magen Biotech), followed by PCR amplification of the 16S rRNA V3–-V4 hypervariable regions with universal primers 338F (5´- ACTCCTACGGGAGGCAGCAG-3´) and 806R (5´- GGACTACHVGGGTWTCTAAT-3´). Amplified libraries were purified using Agencourt AMPure XP beads (Beckman Coulter), quantified via Agilent 2100 Bioanalyzer (Agilent Technologies), and sequenced on the BGISEQ platform (BGI-Shenzhen).

Raw sequencing data underwent rigorous quality control: low-quality reads (Q-score <20), adapter contaminants, and ambiguous sequences (>5% N bases) were filtered using Readfq v.8. Paired-end reads were merged with FLASH (v.1.2.11) using a 10 bp minimum overlap. Chimeric sequences were identified and removed via UCHIME algorithm within USEARCH (v.11.0.667). Operational taxonomic units were clustered at 97% similarity using UPARSE, with taxonomic annotation performed against the Greengenes database (v.13_8) via the RDP classifier (v.2.13) with an 80% confidence threshold. Microbial community structure was visualized through principal coordinates analysis (ade4 package, R v.4.1.2) and hierarchical clustering heatmaps (ggplot2, R v.4.1.2). Species-level compositional profiles were generated to compare microbiota shifts between experimental groups. This integrated workflow ensures high-resolution characterization of wound microbiota ecology, linking taxonomic succession patterns with healing phenotypes through standardized bioinformatics pipelines.

### Bacterial strain activation and culture techniques

#### Strain revival and subculturing

Lyophilized *Prevotella copri* stocks (BeNa Culture Collection, China) were rehydrated with 500 µL anaerobic sterile water within an anaerobic chamber (Coy Laboratory Products). Two hundred microliters of the suspension was spread onto pre-reduced Columbia blood agar plates (Hopebio) using sterile glass beads, followed by anaerobic incubation at 37°C for 5 days (AnaeroPack System, Mitsubishi Gas Chemical). Single colonies were isolated through quadrant streaking and subcultured under identical conditions to ensure purity.

#### Liquid culture protocol

A modified BHI broth was prepared containing 38.5 g/L brain heart infusion (Huankai Microbial, China), 1 g/L L-cysteine hydrochloride (MCE), 10 mg/L hemin chloride (MCE), and 1 mg/L vitamin K1 (MCE). The medium was adjusted to pH 6.9 ± 0.1, aliquoted into anaerobic tubes, and autoclaved at 121°C for 20 min. Primary plate cultures were inoculated into 10 mL broth and incubated statically at 37°C for 72 h under anaerobic conditions. Optical density at 600 nm (OD_600_) was monitored to confirm logarithmic growth phase (OD_600_ = 0.6–0.8).

### Metabolomic profiling of bacterial secretions

#### Metabolites extraction

One hundred microliters of sample was taken, mixed with 400 µL of extraction solution (MeOH:ACN, 1:1 [vol/vol]), which contained deuterated internal standards. The mixed solution was vortexed for 30 s, sonicated for 10 min in a 4°C water bath, and incubated for 1 h at −40°C to precipitate proteins. Then the samples were centrifuged at 12,000 rpm (RCF = 13,800 (× *g*), *R* = 8.6 cm) for 15 min at 4°C. The supernatant was transferred to a fresh glass vial for analysis.

#### LC-MS/MS analysis

For polar metabolites, LC-MS/MS analyses were performed using an UHPLC system (Vanquish, Thermo Fisher Scientific) equipped with a Waters ACQUITY UPLC BEH Amide (2.1 mm × 50 mm, 1.7 µm) column coupled to Orbitrap Exploris 120 mass spectrometer (Thermo Fisher Scientific). The mobile phase consisted of 25 mmol/L ammonium acetate and 25 ammonia hydroxide in water (pH = 9.75) (A) and acetonitrile (B). The auto-sampler temperature was 4°C, and the injection volume was 2 µL. Mass spectrometry detection was operated in information-dependent acquisition (IDA) mode controlled by Xcalibur software (Thermo Fisher Scientific) to acquire MS/MS spectra. In this mode, the software continuously evaluates the full-scan MS spectrum. The ESI source conditions were set as follows: sheath gas flow rate 50 Arb, aux gas flow rate 15 Arb, capillary temperature 320°C, spray voltage 3.8 kV (positive) or −3.4 kV (negative), full MS resolution at 60,000, MS/MS resolution at 15,000. A stepped normalized collision energy (NCE) of 20, 30, and 40 was applied.

#### Data preprocessing and annotation

The raw data were converted to the mzXML format using ProteoWizard and processed with an in-house program, which was developed using R and based on XCMS, for peak detection, extraction, alignment, and integration. The R package and the BiotreeDB (v.3.0) were applied in metabolite identification.

### Transcriptomic sequencing and bioinformatics

Total RNA was extracted from wound tissues using TRIzol (Invitrogen), with quality verified by Bioanalyzer 2100 (RIN > 8.0). Libraries were prepared using NEBNext Ultra II RNA Kit and sequenced on BGISEQ-500. Raw reads were quality-trimmed using Trimmomatic (SLIDINGWINDOW:4:20) and aligned to mm10 genome via STAR (v.2.7.9a). Gene expression quantification employed RSEM (v.1.3.3), with differential expression analysis using DESeq2 (|log2FC| > 1, *P*_adj_ < 0.05). Functional enrichment of DEGs was performed through clusterProfiler (v.4.2.2) using GO databases, with hypergeometric testing and Benjamini-Hochberg correction (FDR < 0.05).

### RNA isolation and quantitative PCR analysis

Total RNA was extracted from wound tissues using TRIzol (Invitrogen) following a standardized phenol-chloroform protocol. Briefly, homogenized tissues underwent phase separation with chloroform, and RNA was precipitated with isopropanol followed by 75% ethanol washes. RNA purity and concentration were quantified via NanoDrop 2000 spectrophotometry (A260/A280 > 1.8, A260/A230 > 2.0; Thermo Fisher Scientific). Reverse transcription of 1 µg total RNA was performed using the PrimeScript RT Reagent Kit (Takara Bio Inc., Japan) with oligo(dT) and random hexamer priming.

qPCR reactions were conducted in 20 µL volumes containing SYBR Green Premix Ex Taq II (Takara Bio) and gene-specific primers (sequences provided in Table S2) on a QuantStudio 7 Flex Real-Time PCR System (Thermo Fisher Scientific). Thermal cycling conditions included initial denaturation (95°C, 30 s), followed by 40 cycles of 95°C (5 s) and 60°C (30 s). Melt curve analysis (60–95°C, 0.3°C/s increments) confirmed primer specificity. Gene expression levels were normalized to 18S rRNA using the 2^−ΔΔCt^ method, with technical triplicates ensuring intra-assay precision.

### Cell viability assay

Cells were seeded in 96-well plates at a density of 5 × 10^3^ cells/well and allowed to adhere for 12 h. After treatment with ceramide (0.781 µM) (Fig. S1), 10 µL CCK-8 reagent (Hanbio Biotechnology) was added to each well, and the plates were subsequently incubated for 3 h. The absorbance was measured at 450 nm with a reference wavelength of 650 nm using a microplate reader (SpectraMax iD5, Molecular Devices). Cell viability was calculated using the following formula: [(A_test_ – A_blank_) / (A_control_ – A_blank_)] × 100%, where A_blank_ represents the absorbance of the blank wells (containing culture medium and CCK-8 reagent only).

## Data Availability

The RNA-seq data files that were generated in this study are available from the NCBI Gene Expression Omnibus (GEO) under the accession number GEO: GSE296532. The original 16S rRNA sequence data are available at the NCBI by accession number PRJNA1259905.

## SUPPLEMENTAL MATERIAL

Fig. S1 spectrum.01587-25-s0001.docx The optimal concentration of ceramide for modulating keratinocyte viability was determined via a CCK-8 screening assay.

Table S1 spectrum.01587-25-s0002.xlsx Metabolome profiling of *Prevotella copri* secretions.

Table S2 spectrum.01587-25-s0003.docx Primers used in this study.

ASM does not own the copyrights to Supplemental Material that may be linked to, or accessed through, an article. The authors have granted ASM a non-exclusive, world-wide license to publish the Supplemental Material files. Please contact the corresponding author directly for reuse.

## References

[1] James GA, Swogger E, Wolcott R, Pulcini ED, Secor P, Sestrich J, Costerton JW, Stewart PS. 2008. Biofilms in chronic wounds. Wound Repair Regen 16:37–44. doi:10.1111/j.1524-475X.2007.00321.x 18086294

[2] Malone M, Bjarnsholt T, McBain AJ, James GA, Stoodley P, Leaper D, Tachi M, Schultz G, Swanson T, Wolcott RD. 2017. The prevalence of biofilms in chronic wounds: a systematic review and meta-analysis of published data. J Wound Care 26:20–25. doi:10.12968/jowc.2017.26.1.20 28103163

[3] Kirketerp-Møller K, Jensen PØ, Fazli M, Madsen KG, Pedersen J, Moser C, Tolker-Nielsen T, Høiby N, Givskov M, Bjarnsholt T. 2008. Distribution, organization, and ecology of bacteria in chronic wounds. J Clin Microbiol 46:2717–2722. doi:10.1128/JCM.00501-08 18508940 PMC2519454

[4] Bjarnsholt T. 2013. The role of bacterial biofilms in chronic infections. APMIS 121:1–58. doi:10.1111/apm.12099 23635385

[5] Grice EA, Segre JA. 2011. The skin microbiome. Nat Rev Microbiol 9:244–253. doi:10.1038/nrmicro2537 21407241 PMC3535073

[6] SanMiguel AJ, Grice EA. 2015. Interactions between host factors and the skin microbiome. Cell Mol Life Sci 72:1499–1515. doi:10.1007/s00018-014-1812-z 25548803 PMC4376244

[7] Byrd AL, Deming C, Cassidy SKB, Harrison OJ, Ng W-I, Conlan S, NISC Comparative Sequencing Program, Belkaid Y, Segre JA, Kong HH. 2017. Staphylococcus aureus and Staphylococcus epidermidis strain diversity underlying pediatric atopic dermatitis. Sci Transl Med 9:eaal4651. doi:10.1126/scitranslmed.aal4651 28679656 PMC5706545

[8] Smith SA, Travers RJ, Morrissey JH. 2015. How it all starts: initiation of the clotting cascade. Crit Rev Biochem Mol Biol 50:326–336. doi:10.3109/10409238.2015.1050550 26018600 PMC4826570

[9] Nurden A. 2011. Platelets, inflammation and tissue regeneration. Thromb Haemost 105:S13–S33. doi:10.1160/THS10-11-0720 21479340

[10] de Oliveira S, Rosowski EE, Huttenlocher A. 2016. Neutrophil migration in infection and wound repair: going forward in reverse. Nat Rev Immunol 16:378–391. doi:10.1038/nri.2016.49 27231052 PMC5367630

[11] Baum CL, Arpey CJ. 2005. Normal cutaneous wound healing: clinical correlation with cellular and molecular events. Dermatol Surg 31:674–686. doi:10.1111/j.1524-4725.2005.31612 15996419

[12] Heng MCY. 2011. Wound healing in adult skin: aiming for perfect regeneration. Int J Dermatology 50:1058–1066. doi:10.1111/j.1365-4632.2011.04940.x 22126865

[13] Xue M, Jackson CJ. 2015. Extracellular matrix reorganization during wound healing and its impact on abnormal scarring. Adv Wound Care 4:119–136. doi:10.1089/wound.2013.0485 PMC4352699 25785236

[14] Barrientos S, Stojadinovic O, Golinko MS, Brem H, Tomic‐Canic M. 2008. Perspective article: Growth factors and cytokines in wound healing. Wound Repair Regen 16:585–601. doi:10.1111/j.1524-475X.2008.00410.x 19128254

[15] Werner S, Grose R. 2003. Regulation of wound healing by growth factors and cytokines. Physiol Rev 83:835–870. doi:10.1152/physrev.2003.83.3.835 12843410

[16] Li J, Zhang Y-P, Kirsner RS. 2003. Angiogenesis in wound repair: angiogenic growth factors and the extracellular matrix. Microsc Res Tech 60:107–114. doi:10.1002/jemt.10249 12500267

[17] Reinke JM, Sorg H. 2012. Wound repair and regeneration. Eur Surg Res 49:35–43. doi:10.1159/000339613 22797712

[18] Gill S, Parks W. 2008. Metalloproteinases and their inhibitors: regulators of wound healing. Int J Biochem Cell Biol 40:1334–1347. doi:10.1016/j.biocel.2007.10.024 18083622 PMC2746915

[19] Eckes B, Nischt R, Krieg T. 2010. Cell-matrix interactions in dermal repair and scarring. Fibrogenesis Tissue Repair 3:4. doi:10.1186/1755-1536-3-4 20222960 PMC2855519

[20] Profyris C, Tziotzios C, Do Vale I. 2012. Cutaneous scarring: pathophysiology, molecular mechanisms, and scar reduction therapeutics Part I. The molecular basis of scar formation. J Am Acad Dermatol 66:1–10; doi:10.1016/j.jaad.2011.05.055 22177631

[21] Greenhalgh DG. 1998. The role of apoptosis in wound healing. Int J Biochem Cell Biol 30:1019–1030. doi:10.1016/S1357-2725(98)00058-2 9785465

[22] Putra I, Rabiee B, Anwar KN, Gidfar S, Shen X, Babalooee M, Ghassemi M, Afsharkhamseh N, Bakhsh S, Missiakas D, Nezamabadi A, Milani B, Eslani M, Djalilian AR. 2019. Staphylococcus aureus alpha-hemolysin impairs corneal epithelial wound healing and promotes intracellular bacterial invasion. Exp Eye Res 181:263–270. doi:10.1016/j.exer.2019.02.019 30822400 PMC6447303

[23] Wang G, Sweren E, Liu H, Wier E, Alphonse MP, Chen R, Islam N, Li A, Xue Y, Chen J, Park S, Chen Y, Lee S, Wang Y, Wang S, Archer NK, Andrews W, Kane MA, Dare E, Reddy SK, Hu Z, Grice EA, Miller LS, Garza LA. 2021. Bacteria induce skin regeneration via IL-1β signaling. Cell Host Microbe 29:777–791. doi:10.1016/j.chom.2021.03.003 33798492 PMC8122070

[24] Enamorado M, Kulalert W, Han S-J, Rao I, Delaleu J, Link VM, Yong D, Smelkinson M, Gil L, Nakajima S, Linehan JL, Bouladoux N, Wlaschin J, Kabat J, Kamenyeva O, Deng L, Gribonika I, Chesler AT, Chiu IM, Le Pichon CE, Belkaid Y. 2023. Immunity to the microbiota promotes sensory neuron regeneration. Cell 186:607–620. doi:10.1016/j.cell.2022.12.037 36640762 PMC11512587

[25] Constantinides MG, Link VM, Tamoutounour S, Wong AC, Perez-Chaparro PJ, Han S-J, Chen YE, Li K, Farhat S, Weckel A, Krishnamurthy SR, Vujkovic-Cvijin I, Linehan JL, Bouladoux N, Merrill ED, Roy S, Cua DJ, Adams EJ, Bhandoola A, Scharschmidt TC, Aubé J, Fischbach MA, Belkaid Y. 2019. MAIT cells are imprinted by the microbiota in early life and promote tissue repair. Science 366:eaax6624. doi:10.1126/science.aax6624 31649166 PMC7603427

[26] Linehan JL, Harrison OJ, Han S-J, Byrd AL, Vujkovic-Cvijin I, Villarino AV, Sen SK, Shaik J, Smelkinson M, Tamoutounour S, Collins N, Bouladoux N, Dzutsev A, Rosshart SP, Arbuckle JH, Wang C-R, Kristie TM, Rehermann B, Trinchieri G, Brenchley JM, O’Shea JJ, Belkaid Y. 2018. Non-classical immunity controls microbiota impact on skin immunity and tissue repair. Cell 172:784–796. doi:10.1016/j.cell.2017.12.033 29358051 PMC6034182

[27] Naik S, Bouladoux N, Linehan JL, Han S-J, Harrison OJ, Wilhelm C, Conlan S, Himmelfarb S, Byrd AL, Deming C, Quinones M, Brenchley JM, Kong HH, Tussiwand R, Murphy KM, Merad M, Segre JA, Belkaid Y. 2015. Commensal–dendritic-cell interaction specifies a unique protective skin immune signature. Nature 520:104–108. doi:10.1038/nature14052 25539086 PMC4667810

[28] Iwase T, Uehara Y, Shinji H, Tajima A, Seo H, Takada K, Agata T, Mizunoe Y. 2010. Staphylococcus epidermidis Esp inhibits Staphylococcus aureus biofilm formation and nasal colonization. Nature 465:346–349. doi:10.1038/nature09074 20485435

[29] Nakamura K, O’Neill AM, Williams MR, Cau L, Nakatsuji T, Horswill AR, Gallo RL. 2020. Short chain fatty acids produced by Cutibacterium acnes inhibit biofilm formation by Staphylococcus epidermidis. Sci Rep 10. doi:10.1038/s41598-020-77790-9 PMC7718897 33277548

[30] Lai Y, Di Nardo A, Nakatsuji T, Leichtle A, Yang Y, Cogen AL, Wu Z-R, Hooper LV, Schmidt RR, von Aulock S, Radek KA, Huang C-M, Ryan AF, Gallo RL. 2009. Commensal bacteria regulate Toll-like receptor 3–dependent inflammation after skin injury. Nat Med 15:1377–1382. doi:10.1038/nm.2062 19966777 PMC2880863

[31] Di Domizio J, Belkhodja C, Chenuet P, Fries A, Murray T, Mondéjar PM, Demaria O, Conrad C, Homey B, Werner S, Speiser DE, Ryffel B, Gilliet M. 2020. The commensal skin microbiota triggers type I IFN–dependent innate repair responses in injured skin. Nat Immunol 21:1034–1045. doi:10.1038/s41590-020-0721-6 32661363

[32] Williams MR, Costa SK, Zaramela LS, Khalil S, Todd DA, Winter HL, Sanford JA, O’Neill AM, Liggins MC, Nakatsuji T, Cech NB, Cheung AL, Zengler K, Horswill AR, Gallo RL. 2019. Quorum sensing between bacterial species on the skin protects against epidermal injury in atopic dermatitis. Sci Transl Med 11:eaat8329. doi:10.1126/scitranslmed.aat8329 31043573 PMC7106486

[33] Dimitriu PA, Iker B, Malik K, Leung H, Mohn WW, Hillebrand GG. 2019. New insights into the intrinsic and extrinsic factors that shape the human skin microbiome. mBio 10:e00839-19. doi:10.1128/mBio.00839-19 31266865 PMC6606800

[34] Zheng Y, Hunt RL, Villaruz AE, Fisher EL, Liu R, Liu Q, Cheung GYC, Li M, Otto M. 2022. Commensal Staphylococcus epidermidis contributes to skin barrier homeostasis by generating protective ceramides. Cell Host Microbe 30:301–313. doi:10.1016/j.chom.2022.01.004 35123653 PMC8917079

[35] Mohammedsaeed W, Cruickshank S, McBain AJ, O’Neill CA. 2015. Lactobacillus rhamnosus GG lysate increases re-epithelialization of keratinocyte scratch assays by promoting migration. Sci Rep 5:16147. doi:10.1038/srep16147 26537246 PMC4633615

[36] Yang Y, Huang J, Zeng A, Long X, Yu N, Wang X. 2024. The role of the skin microbiome in wound healing. Burns Trauma 12:tkad059. doi:10.1093/burnst/tkad059 38444635 PMC10914219

[37] Canchy L, Kerob D, Demessant A, Amici JM. 2023. Wound healing and microbiome, an unexpected relationship. J Eur Acad Dermatol Venereol 37 Suppl 3:7–15. doi:10.1111/jdv.18854 36635613

[38] Round JL, Lee SM, Li J, Tran G, Jabri B, Chatila TA, Mazmanian SK. 2011. The Toll-like receptor 2 pathway establishes colonization by a commensal of the human microbiota. Science 332:974–977. doi:10.1126/science.1206095 21512004 PMC3164325

[39] Li Y, Nicholson RJ, Summers SA. 2022. Ceramide signaling in the gut. Mol Cell Endocrinol 544:111554. doi:10.1016/j.mce.2022.111554 34998898 PMC8828712

[40] Brown EM, Ke X, Hitchcock D, Jeanfavre S, Avila-Pacheco J, Nakata T, Arthur TD, Fornelos N, Heim C, Franzosa EA, Watson N, Huttenhower C, Haiser HJ, Dillow G, Graham DB, Finlay BB, Kostic AD, Porter JA, Vlamakis H, Clish CB, Xavier RJ. 2019. Bacteroides-derived sphingolipids are critical for maintaining intestinal homeostasis and symbiosis. Cell Host Microbe 25:668–680. doi:10.1016/j.chom.2019.04.002 31071294 PMC6544385

[41] Håversen L, Danielsson KN, Fogelstrand L, Wiklund O. 2009. Induction of proinflammatory cytokines by long-chain saturated fatty acids in human macrophages. Atherosclerosis 202:382–393. doi:10.1016/j.atherosclerosis.2008.05.033 18599066

[42] Scott NA, Andrusaite A, Andersen P, Lawson M, Alcon-Giner C, Leclaire C, Caim S, Le Gall G, Shaw T, Connolly JPR, Roe AJ, Wessel H, Bravo-Blas A, Thomson CA, Kästele V, Wang P, Peterson DA, Bancroft A, Li X, Grencis R, Mowat AM, Hall LJ, Travis MA, Milling SWF, Mann ER. 2018. Antibiotics induce sustained dysregulation of intestinal T cell immunity by perturbing macrophage homeostasis. Sci Transl Med 10:2561. doi:10.1126/scitranslmed.aao4755 PMC6548564 30355800

[43] Gilliver SC. 2010. Sex steroids as inflammatory regulators. J Steroid Biochem Mol Biol 120:105–115. doi:10.1016/j.jsbmb.2009.12.015 20045727

