# Supplementary material for: Prevotella copri facilitates wound healing in mice through the sphingosine-CerS1-ceramide metabolic pathway
Source: Microbiol Spectr. 2025 Nov 14;14(1):e01587-25. doi: 10.1128/spectrum.01587-25 (PMC12772305; doi:10.1128/spectrum.01587-25)
Supplement: Fig. S1 — The optimal concentration of ceramide for modulating keratinocyte viability was determined via a CCK-8 screening assay. [file spectrum.01587-25-s0001.docx]

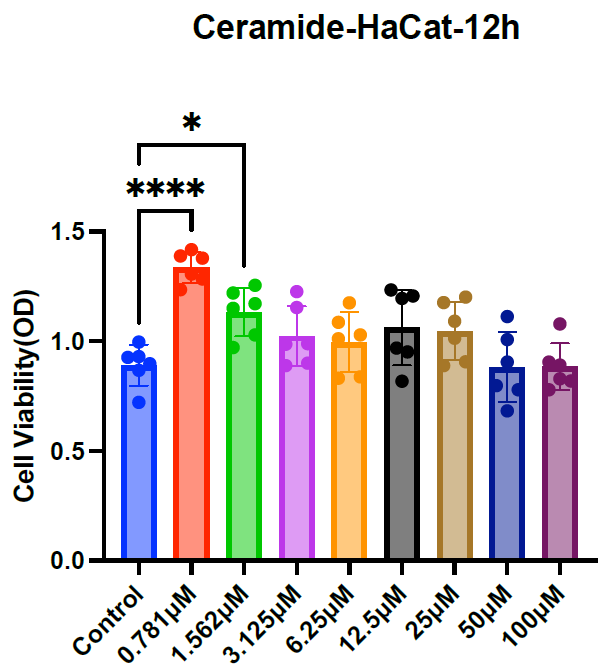


**Fig S1. The optimal concentration of ceramide for modulating keratinocyte viability was determined via a CCK-8 screening assay.**
