# Supplementary material for: Prevotella copri facilitates wound healing in mice through the sphingosine-CerS1-ceramide metabolic pathway
Source: Microbiol Spectr. 2025 Nov 14;14(1):e01587-25. doi: 10.1128/spectrum.01587-25 (PMC12772305; doi:10.1128/spectrum.01587-25)
Supplement: Table S2 — Primers used in this study. [file spectrum.01587-25-s0003.docx]

**Supplementary Table 2.** List of Primers used in this study

| Primers | Sequence (5' to 3') |
| --- | --- |
| CerS-1-F | TTCCGGTACCACAACGTAGG |
| CerS-1-R | GTAGAGGCGGAACCAGAACC |
| CerS-2-F | CTAGAAGTGGGAAACGGAGTAGC |
| CerS-2-R | AAGTTCACAGGCAGCCATAGT |
| CerS-3-F | CTTAGGATTCGGCAGAAGCA |
| CerS-3-R | ACAGCTCCAGCCATGGTTAT |
| CerS-4-F | CTGGGCTTCTACCTTTCACTGC |
| CerS-4-R | AATGCGGAGCAAGTTGACGCTG |
| CerS-5-F | CATCGGAGGAATCAGGACAAG |
| CerS-5-R | ACCAAGGCATCGACCAGAGA |
| CerS-6-F | CAGACCTGAAGAACACGGAGGA |
| CerS-6-R | GTCCATTGGCTTGGATGTTGAGG |
| Has-1-F | CTTTCAAGGCACTGGGCGAC |
| Has-1-R | CACCGCTTCATAGGTCATCC |
| Has-2-F | ACAGGCACCTTACCAACAGGGTGT |
| Has-2-R | GCATGCATAGATCAAAGTTCCCACG |
| Has-3-F | ACTGCCTTCAAGGCCCTTGG |
| Has-3-R | AATGTTCCAGATGCGGCCAC |
| Hyal-1-F | GCCCTTCAGTCCTGAGGTTT |
| Hyal-1-R | ACAGTGATGAATGGCCGGTT |
| Hyal-2-F | ACTGAGCCAGGTGGACCTTA |
| Hyal-2-R | GAGTCGCCCCAGAAGATGAC |
| 18S-F | TCAACTTCGATGGTAAAGGTTG |
| 18S-R | GAGGAGTTCATCGGTTCGAA |
